# Community Participation in Habitat Management and Larviciding for the Control of Malaria Vectors in Southern Malawi

**DOI:** 10.4269/ajtmh.21-1127

**Published:** 2022-11-21

**Authors:** Steven Gowelo, Paola Meijer, Tinashe Tizifa, Tumaini Malenga, Monicah M. Mburu, Alinune N. Kabaghe, Dianne J. Terlouw, Michèle van Vugt, Kamija S. Phiri, Themba Mzilahowa, Constantianus J.M. Koenraadt, Henk van den Berg, Lucinda Manda-Taylor, Robert S. McCann, Willem Takken

**Affiliations:** ^1^ School of Global and Public Health, Kamuzu University of Health Sciences, Blantyre, Malawi;; ^2^ Laboratory of Entomology, Wageningen University & Research, Wageningen, The Netherlands;; ^3^ MAC Communicable Diseases Action Centre, Kamuzu University of Health Sciences, Blantyre, Malawi;; ^4^ Academic Medical Centre, University of Amsterdam, Amsterdam, The Netherlands;; ^5^ African Institute for Development Policy, Lilongwe, Malawi;; ^6^ Macha Research Trust, Choma, Zambia;; ^7^ Liverpool School of Tropical Medicine, Liverpool, United Kingdom;; ^8^ Center for Vaccine Development and Global Health, University of Maryland School of Medicine, Baltimore, Maryland

## Abstract

Larval source management (LSM) could reduce malaria transmission when executed alongside core vector control strategies. Involving communities in LSM could increase intervention coverage, reduce operational costs, and promote sustainability via community buy-in. We assessed the effectiveness of community-led LSM to reduce anopheline larval densities in 26 villages along the perimeter of Majete Wildlife Reserve in southern Malawi. The communities formed LSM committees which coordinated LSM activities in their villages following specialized training. Effectiveness of larviciding by LSM committees was assessed via pre- and post-spray larval sampling. The effect of community-led LSM on anopheline larval densities in intervention villages was assessed via comparisons with densities in non-LSM villages over a period of 14 months. Surveys involving 502 respondents were undertaken in intervention villages to explore community motivation and participation, and factors influencing these outcomes. Larviciding by LSM committees reduced anopheline larval densities in post-spray sampling compared with pre-spray sampling (*P* < 0.0001). No differences were observed between anopheline larval densities during pre-spray sampling in LSM villages and those in non-LSM villages (*P* = 0.282). Knowledge about vector biology and control, and someone’s role in LSM motivated community participation in the vector control program. Despite reducing anopheline larval densities in LSM villages, the impact of the community-led LSM could not be detected in our study setting because of low mosquito densities after scale-up of core malaria control interventions. Still, the contributions of the intervention in increasing a community’s knowledge of malaria, its risk factors, and its control methods highlight potential benefits of the approach.

## INTRODUCTION

In recent years, there has been renewed interest in larval source management (LSM) as a complementary tool for malaria control in Africa.^1^^,^^2^ LSM has contributed to reductions in adult vector populations^3^ and malaria burden, especially where it has been integrated with other vector control tools.^4^ Currently, LSM remains less widely adopted for malaria control in many African countries often because of a knowledge gap in local larval mosquito vector ecology,^5^^,^^6^ concerns about inefficiency of ground applications of nonresidual larvicides^7^ and costs of large-scale implementation,^8^^,^^9^ and lack of local evidence of its impact in malaria control.^10^

The two most common types of LSM are habitat modification and larviciding. Habitat modification involves physical manipulation of a larval habitat through draining, filling, and land leveling.^11^ This could be considered a more sustainable method as habitats are permanently disrupted to become unfavorable for mosquito breeding. Larviciding is usually done by application of an endotoxin-producing bacterial larvicide, *Bacillus thuringiensis* var. *israelensis* (*Bti*), rather than synthetic insecticides.^12^^–^^15^ Although *Bti* is target species–specific due to the presence of membrane receptors in dipteran insects, poses no harm to the environment, has reduced probability for development of resistance^16^^–^^25^ and is cost-effective,^16^^,^^26^ larviciding in areas with numerous breeding sites is logistically demanding.^13^^,^^27^

Wherever LSM has been implemented, vertical management approaches have had little community involvement.^28^^–^^31^ There is growing recognition of the need for partnerships between experts and communities in vector management^27^^,^^32^^–^^38^ to ensure sustainability^6^^,^^34^ and increased intervention acceptability and uptake.^39^^,^^40^ Nevertheless, it remains unclear whether community-led LSM is feasible and effective in reducing anopheline larval densities in rural areas. Here we assessed community motivation and participation in *Bti* larviciding and habitat management, and effect on anopheline larval densities. This will provide evidence on the potential of community-led LSM in reducing the malaria risk in settings with numerous aquatic habitats.

## METHODS

### Study area.

**Figure 1.**
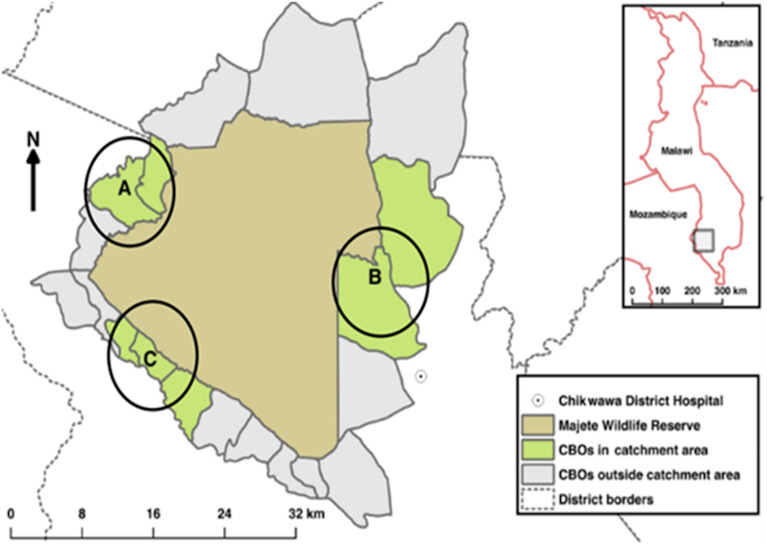
Map of Majete Wildlife Reserve and the Majete Perimeter showing the three focal areas.

The study was conducted in 46 villages along the perimeter of the Majete Wildlife Reserve in Chikwawa District (16°1′S; 34° 47′E), southern Malawi, as part of a community-led malaria control research program, Majete Malaria Project (MMP). The MMP was implemented in 65 villages along the perimeter of the wildlife reserve,^37^^,^^38^ divided into three subregions called focal areas A, B, and C (Figure 1).^41^ As a cluster randomized control trial to assess the additive effects of community-based house improvement (HI) and LSM to the interventions of the Malawi National Malaria Control Program, village clusters under MMP were randomly allocated to one of four trial arms: 1) a control arm, 2) HI, 3) LSM, and 4) HI+LSM. The Malawi National Malaria Control Program interventions and community engagement were applied in all arms. To reduce contamination risk between trial arms, each cluster was separated from the others by at least 800 m.^42^ In the current study, we sought to assess community motivation and participation in *Bti* larviciding and habitat management, and the effect on anopheline larval densities; hence only LSM villages (i.e., LSM and LSM+HI) were selected. These villages are, in this study, collectively referred to as LSM or intervention villages, and the remaining villages are referred to as non-LSM villages. Effectively, the study was conducted in 26 LSM and 20 non-LSM villages.

Chikwawa district is predominately rural, with most people in the study area raising livestock, including cattle, goats and pigs, and practicing subsistence farming, cultivating predominantly maize and millet. The district is generally hot and dry from September to December, hot and rainy from January to April, and dry with mild temperatures from June to August. Most of the terrain in the study area is flat and lies within two river valleys situated alongside hilly, highland areas along the north and west of the study area. The availability of water, especially in the rainy season, and prolonged high temperatures create favorable humid conditions for mosquitoes in the area. There is a wide variety of potential larval habitats for anophelines in the area, including dams, swamps, ponds, borehole runoffs and drainage channels.^43^ The malaria vectors *Anopheles gambiae s.s.*, *An. arabiensis*, and *An. funestus* are all present in Chikwawa district.^18^

### Description of community-led activities.

In this study, LSM activities were conducted by local communities. As part of MMP, all 65 villages in the study area were part of an intensive community engagement program focusing on community workshops led by trained volunteers called health animators (HAs).^44^ One or two HAs (based on village size) were selected by village heads and members of the community to coordinate the local malaria control initiatives.^37^ Before commencement of their work, the HAs received training from the MMP research team in collaboration with The Hunger Project—Malawi (THP), Ministry of Health (Chikwawa District Health Office), and African Parks—Majete on malaria-related topics on a tailored curriculum.

In LSM villages, the HAs received additional training specific to LSM. Together with village heads and other community leaders, the HAs facilitated the selection of groups of between 10 to 12 volunteers per LSM village to form “LSM committees.” These groups were tasked with coordinating all the LSM activities in their respective communities, including mapping of all potential mosquito larval habitats in their villages, facilitating draining and filling of habitats, planning and conducting weekly *Bti-*treatment of all potential habitats holding water in their villages, and reporting to both the MMP research team (through field supervisor) and their communities via written reports and village workshops, respectively. The general community assisted each LSM committee with larval habitat management through draining or filling potential larval habitats.

**Figure 2.**
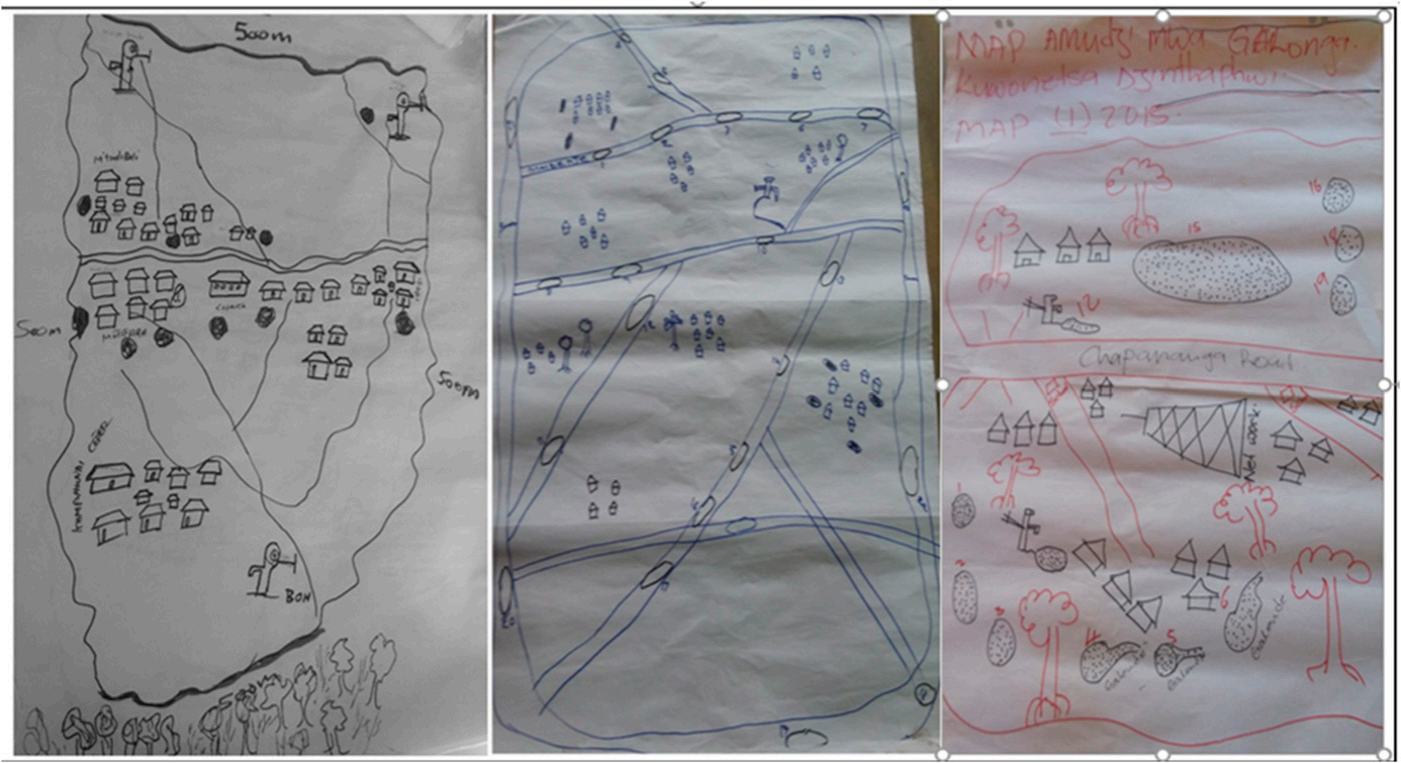
Community participatory mapping of potential mosquito breeding sites.

In May 2016, before the LSM activities, the LSM committees received trainings focused on 1) the role of mosquitoes in malaria transmission; 2) recognizing mosquito larvae; 3) the biology of mosquitoes; 4) mosquito larval habitats; 5) control of mosquitoes via disruption of larval habitats; 6) habitat draining, filling, and larviciding as control tools; 7) activity of *Bti* as a larvicide; 8) operation of spraying machines; 9) *Bti-*water measurements and spraying; 10) tracking longitudinal changes on numbers and sizes of habitats containing water over time; 11) intervention evaluation; 12) activity reporting; and 13) planning. The training sessions incorporated many practical aspects where the participants were first introduced to the parts of a sprayer and its assemblage followed by preparation of *Bti-*water mixtures following guidelines by the larvicide manufacturers. The training sessions also involved actual spraying of mosquito larval habitats with predetermined amounts of *Bti* based on the surface area of the water body. Assessment of the efficacy of the spraying activities and reporting to the research team and their communities were the last pieces of the training sessions. To manage the activities effectively and cover all potential mosquito larval habitats, the LSM committees, in liaison with community members and HAs, developed work plans and drew maps of their villages detailing all the potential habitats (Figure 2). Habitat-tracking forms were developed to guide the committees in tracking habitat presence and size, and also to the spraying activities’ efficacy on larval densities. After completion by the LSM committees, copies of the habitat-tracking forms were sent to a THP project officer who later forwarded them to the MMP field supervisor.

### Evaluation of community-led larviciding.

To assess the effect of community-led *Bti-*treatment on anopheline larval densities, the MMP research team conducted independent larval density sampling surveys in *Bti-*treated water bodies in LSM villages, as well as in water bodies in non-LSM villages. In these surveys, three water bodies per village were selected using a “spin-the-bottle” method (Supplemental Appendix 1) every 2 to 3 months (termed a “round”). The timing of each round was set to enable the research team to carry out other project activities and did not affect the weekly larviciding activities by LSM committees. Because of dry spells prevalent during the study period, no or fewer than three habitats were effectively visited in some villages. Because of the scarcity of water-containing habitats, seven non-LSM villages did not have any habitats to sample throughout the four surveys. Five rounds of anopheline larval density surveys over 14 months (April 2017–May 2018) were undertaken in LSM villages, whereas four rounds over 11 months (July 2017–May 2018) were conducted in the non-LSM villages. In round 1, the larval density sampling was conducted in LSM villages only, whereas rounds 2 through 5 were undertaken in LSM and non-LSM villages.

For each larval density survey, every selected larval habitat in the LSM villages had a pre-spray survey done at least 4 days after the previous *Bti* application and either 1, 2, or 3 days before the next *Bti* application, and a post-spray survey done 2 or 3 days after the last application of *Bti.* The two surveys would respectively establish whether any larvae were present in the habitat before *Bti* was applied and determine whether the *Bti* effectively killed the larvae in the habitat. Because there was no *Bti* treatment in non-LSM villages, larval density surveys in selected habitats did not include post-spray surveys. We assumed that consistent application of *Bti* would suppress anopheline larval densities over time in LSM villages and hence enable comparisons of pre-spray larval densities in LSM villages with larval densities in non-LSM villages.

The number of larval sampling points at each habitat was dependent on the habitat’s perimeter. One sampling point was selected for habitats with perimeters ≤ 10 m. Two and three samples were taken at habitats with perimeters > 10 m but < 30 m and > 30 m, respectively. For each sample, a circular aluminum tin, open on both ends, 27 cm in diameter and 45 cm high was used. This “area sampler” has been shown to be effective for sampling of larvae.^45^ Within potential larval habitats, the area samplers were placed in positions with the following characteristics: 1) water did not exceed 30 cm; 2) locations arrived at after evenly dividing habitat perimeter by the number of sampling points, except for habitats with perimeters ≤ 10 m; and 3) at least 75% of the area sampler covered with water. All mosquito larvae and pupae were collected from within the area sampler using a 300-mL dipper, fish net, and pipette. The collected larvae were sorted by subfamily, anopheline, or culicine. All anopheline larvae were further sorted into separate instar stages. The sum of all anopheline larvae collected per area sampler divided by the number of samples taken for the habitat on the same day yielded the anopheline larval density for each habitat.^43^ Each habitat was geo-referenced during sampling. For each larval habitat, data were collected on water depth, permanence, and presence of aquatic vegetation. Water depth was reported as an average from three measurements performed at randomly selected positions along the edges and in the center of the larval habitat. All the data were recorded on an Open Data Kit (ODK) form uploaded on a tablet.

### Knowledge, attitude, and practice survey of communities involved in larval control of malaria vectors.

A knowledge, attitude, and practice (KAP) survey was conducted to understand the factors enhancing or hampering community participation in the LSM activities with community members from the 26 LSM villages. This survey was not conducted in the non-LSM villages. Data were collected through a standard structured questionnaire developed in English and uploaded on a Samsung tablet (Supplemental Appendix 2). Two groups of respondents were enrolled in the study: 1) HAs and LSM committee members, who had all received training directly from the project, and 2) members from the general community. For each LSM village, participants were systematically selected from a randomized list of household heads. Any household member present at the time who was older than 18 years was asked to participate. If eligible participants were not available or present in a selected household, an eligible participant was sought from the nearest neighboring house. To ensure sufficient representation of the HAs and LSM committee members in the study, five LSM committee members and all HAs from each LSM village (*N* = 149 and *N* = 25, respectively) were included in the interviews. The tablet-based question guides were administered by trained research assistants in the local language, Chichewa, and entered in English. The question guides included questions on demographic features, knowledge on malaria, mode of transmission, symptoms, possibilities for vector control and methods, and motivation and participation in LSM activities. Before data collection commenced, 1 day of training was conducted for research assistants to familiarize themselves with the questionnaire. After this, a 1-day field pilot was organized to practice the questionnaires in a real-life setting and adjust the questions as needed.

### Ethical statement.

The KAP survey was carried out in conformity with the principles of human subjects’ protection. Ethical approval was obtained from the College of Medicine Research and Ethics Committee (protocol number P.12/17/2222). Before data collection activities commenced, key gatekeepers were sensitized and informed on the purpose of the study. Permission was sought from chiefs for entry into the communities. The participants were clearly informed on the purpose of the study and the potential risks and benefits of participating in it. The participants were further informed about their rights to participate in the research, including the right to refuse or withdraw from participation without negative consequences. Written informed consent was obtained from all participants during data collection.

### Data analysis.

Zero-inflated negative binomial (ZINB) models accounting for both over-dispersion and excess zeros in the data were fitted to elucidate differences in anopheline larval densities between non-LSM and LSM villages, and during pre- and post-*Bti* spray surveys in LSM villages. The negative binomial component was fitted with a log link, and the zero-inflated component was fitted with a logit link.

For the KAP survey data, the responses to the questionnaire’s open-ended questions were coded after completion of the survey. χ^2^ tests were used to examine whether the distribution of individuals among the categories of one variable was independent of their distribution among the categories of another. Multivariable binary logistic regression analyses of participants’ responses and characteristics were used in a backward stepwise approach to explain variations in respondents’ motivation and participation in the community-led LSM. The automated step methods reduce the number of independent variables in a model to come up with a smaller but effective set of independent variables. All data were analyzed using SPSS version 20 (SPSS, Inc., Chicago, IL).

## RESULTS

### Anopheline larval densities.

Between April 2017 and May 2018, the research team made 561 visits to potential larval habitats in 39 villages to monitor anopheline larval densities. In total, 251 habitats in LSM villages were visited for pre-spray surveys with 214 of these revisited for post-spray surveys. An additional 96 habitats were visited in non-LSM villages. Seven of the 20 non-LSM villages included in the study did not have water-filled habitats during the larval sampling periods.

The densities of anopheline larvae in pre-spray and post-spray sampling in LSM villages ranged from 0 to 46 (median = 0) and 0 to 23 (median = 0), respectively. On the basis of the ZINB model, fewer anopheline larvae were observed in post-spray surveys compared with the corresponding pre-spray surveys across the five rounds of sampling (*P* < 0.0001) (Table 1). Compared with the other rounds (i.e., 2–5), significantly more anopheline larvae were collected in round 1 (*P* < 0.0001).

**Table 1:** Summary results of the ZINB model and the effects of the different variables on anopheline larval densities in LSM villages

Variable	Coef.	SE	95% CI		Z	*P* value
			Lower	Upper		
Round						
1						
2	−4.17348	0.748637	−5.64078	−2.70618	−5.57	**<0.0001**
3	−4.77879	0.835139	−6.41564	−3.14195	−5.72	**<0.0001**
4	−5.52657	0.928054	−7.34552	−3.70762	−5.96	**<0.0001**
5	−4.68364	0.753156	−6.1598	−3.20748	−6.22	**<0.0001**
Survey type						
Pre						
Post	−2.11744	0.597716	−3.28894	−0.94594	−3.54	**<0.0001**
Constant	4.747347	1.110279	2.57124	6.923454	4.28	**<0.0001**
Variables explaining zero inflation						
Constant	−25.8355	228307.2	−447500	447448.1	0	1
ln α	2.807609	0.183539	2.44788	3.167338	15.3	**<0.0001**
α	16.57025	3.04128	11.5638	23.74419		

Coef. = coefficient; LSM = larval source management; SE = standard error; ZINB = zero-inflated negative binomial.

*P* values in bold are significant at alpha = 0.05.

The ZINB model showed no significant differences in anopheline larval densities between the LSM and non-LSM villages during the pre-spray surveys in rounds 2 to 5 (*P =* 0.282) (Table 2). The median value of larval densities per round was zero in both LSM and non-LSM villages (range: 0–2.25).

**Table 2:** Summary results of the ZINB model and the effects of the different variables on anopheline larval densities in LSM and non-LSM villages

Variable	Coef.	SE	95% CI		Z	*P* value
			Lower	Upper		
Round						
2						
3	−0.67728	0.989127	−2.61594	1.26137	−0.68	0.494
4	−1.48787	1.282507	−4.00154	1.025799	−1.16	0.246
5	0.45203	0.686627	−0.89373	1.797794	0.66	0.51
Intervention arm						
LSM						
Non-LSM	−0.66428	0.617747	−1.87505	0.546478	−1.08	0.282
Constant	−2.35765	0.996688	−4.31112	−0.40417	−2.37	**0.018**
Variables explaining zero inflation						
Constant	−27.1811	419524.7	−822281	822226.1	0	1
ln α	−1.88327	5.984252	−13.6122	9.845654	−0.31	0.753
α	0.152093	0.910162	1.23E-06	18876.14		

Coef. = coefficient; LSM = larval source management; SE = standard error; ZINB = zero-inflated negative binomial.

Here only pre-spray data from LSM villages were used and these were compared to results from non-LSM villages. *P* values in bold are significant at alpha = 0.05.

### Community KAP in malaria control via LSM.

#### Demographic characteristics.

A total of 502 participants participated in the KAP study conducted in LSM villages only. The majority of participants, 44.2%, belonged to the 26- to 40-year age group (Table 3). Most of the participants were female (60.8%), had primary school education (55.8%), and were engaged in subsistence farming as their primary occupation (67.5%). HA+LSM committee members were younger, more educated and less likely to be unemployed compared with community participants (Table 3).

**Table 3:** Summary of socio-demographic characteristics of the study participants from LSM villages

Characteristics	Participants from the general community	HAs+LSM committee members
Total participants	328	174
Gender (χ^2^ = 3.483; *df* = 3; *P* = 0.062)		
Male	119 (36.3)	78 (44.8)
Female	209 (63.7)	96 (55.2)
Age (χ^2^ = 18.261; *df* = 3; *P* < 0.001)		
18–25	94 (28.7)	47 (27.0)
26–40	126 (38.4)	96 (55.2)
41–64	81 (24.7)	27 (15.5)
65+	27 (8.2)	4 (2.3)
Education (Fisher’s exact = 13.062; *P* = 0.002)		
None	99 (30.2)	28 (16.1)
Primary	171 (52.1)	109 (62.6)
Secondary	57 (17.4)	37 (21.3)
Tertiary	1 (0.3)	0 (0.0)
Occupation (Fisher’s exact = 9.958; *P* = 0.039)		
None	19 (5.8)	3 (1.7)
Manual labor	51 (15.5)	23 (13.2)
Farmer	214 (65.2)	125 (71.8)
Business	33 (10.1)	22 (12.6)
Formal employment	11 (3.4)	1 (0.6)

HA = health animator; LSM = larval source management.

The χ^2^ tests indicate comparisons between the participants from the general community and HAs+LSM committee members.

### Perceived susceptibility of malaria.

On the basis of responses about whom they perceived to be most at risk of malaria, 59.6% and 55.6% of participants from the general community and HAs+LSM committee members, respectively, cited children aged under 5 years (Table 4). Pregnant women were considered the second most susceptible group, with 22% and 28% of participants from the general community and HAs+LSM committee members, respectively, mentioning this risk group.

**Table 4:** People perceived to be the most at risk of malaria by study participants from LSM villages

Characteristic	Frequency of responses (%)		Statistic
	Participants from the general community	HAs+LSM committee members	
Total number of responses	403	257	
Children < 5	240 (59.6)	143 (55.6)	**χ^2^ = 5.107; *df* = 1; *P* = 0.024**
Youth < 15	1 (0.2)	1 (0.4)	χ^2^ = 0.209; *df* = 1; *P* = 0.648
Women	8 (2.0)	1 (0.4)	χ^2^ = 2.244; *df* = 1; *P* = 0.134
Pregnant women	73 (18.1)	72 (28.0)	**χ^2^ = 20.240; *df* = 1; *P* < 0.001**
Adults	1 (0.2)	1 (0.4)	χ^2^ = 0.209; *df* = 1; *P* = 0.648
Elderly	11 (2.7)	6 (2.3)	χ^2^ = 0.003; *df* = 1; *P* = 0.956
Farmers	2 (0.5)	3 (1.2)	χ^2^ = 1.432; *df* = 1; *P* = 0.231
Those not using preventive measures	15 (3.7)	4 (1.6)	χ^2^ = 1.615; *df* = 1; *P* = 0.204
Those with a weak immune system or AIDS	2 (0.5)	2 (0.8)	χ^2^ = 0.419; *df* = 1; *P* = 0.517
Those living close to mosquito aquatic habitats	2 (0.5)	0 (0.0)	χ^2^ = 1.065; *df* = 1; *P* = 0.302
Nonresidents	0 (0.0)	13 (5.1)	**χ^2^ = 25.157; *df* = 1; *P* < 0.001**
Anyone	45 (11.2)	10 (3.9)	**χ^2^ = 7.407; *df* = 1; *P* = 0.006**
No idea	3 (0.7)	1 (0.4)	χ^2^ = 0.166; *df* = 1; *P* = 0.648

HA = health animator; LSM = larval source management.

The χ^2^ tests indicate comparisons between participants from the general community and HAs+LSM committee members. *P* values in bold are significant at alpha = 0.05.

### Knowledge of mode of spread of malaria.

Mosquito bites were mentioned by 98% of respondents as the mode of malaria transmission. Fly bites, soaking in rain, witchcraft, and no idea were incorrect responses from the remaining 2% of respondents. The two respondent groups’ responses were not significantly different, (χ^2^ = 0.626, *df* = 1, *P =* 0.429) in this respect.

Of the 98% of respondents who mentioned mosquito bites as the mode of spread of malaria, 58% correctly mentioned female anophelines as the vector. Differences in this knowledge were observed between the two respondent groups (χ^2^ = 99.129; *df* = 2; *P <* 0.001) with more HAs+LSM committee members (86.8%) than participants from the general community (43.3%) providing the correct answer.

### Knowledge of mosquito larvae.

Significantly more HAs+LSM committee members mentioned their ability to recognize mosquito larvae (95.9%) than participants from the general community (31%) (χ^2^ = 194.117; *df* = 1; *P <* 0.001). Further, significantly more HAs+LSM committee members (90.2%) than members from the general community (0%) mentioned being able to distinguish culicine from anopheline larvae: χ^2^ =431.838, *df* = 1, *P <* 0.001.

### Knowledge of mosquito larval habitats.

When asked where mosquito breeding takes place in their communities, many potential larval habitat types were cited (Table 5). Interestingly, human-made habitats such as borehole runoffs, dams, brick pits, wells, and pit latrines were the most mentioned larval habitats. Ordered in terms of frequency of responses, borehole runoffs and dams were most often mentioned. Wells and pit latrines were the second and third most mentioned habitat types, even though the latter are generally not considered anopheline breeding sites.

**Table 5:** Knowledge of anopheline breeding habitats by study participants from LSM villages

Characteristic	Frequency of responses (%)		Statistic
	Participants from the general community	HAs+LSM committee members	
Total responses	767	466	
Pit latrine	79 (10.3)	45 (9.7)	χ^2^ = 0.193; *df* = 1; *P* = 0.660
Rice paddies	4 (0.5)	0 (0.0)	χ^2^ = 2.139; *df* = 1; *P* = 0.144
Wells	83 (10.8)	63 (13.5)	**χ^2^ = 6.551; *df* = 1; *P* = 0.010**
Drainage channels	30 (3.9)	12 (2.6)	χ^2^ = 0.751; *df* = 1; *P* = 0.386
Borehole run-offs	133 (17.3)	47 (10.1)	**χ^2^ = 9.059; *df* = 1; *P* = 0.003**
Dams	97 (12.6)	72 (15.5)	**χ^2^ = 7.096; *df* = 1; *P* = 0.008**
Stream beds	59 (7.7)	42 (9.0)	χ^2^ = 2.676; *df* = 1; *P* = 0.102
Freshwater marshes	67 (8.7)	35 (7.5)	χ^2^ = 0.007; *df* = 1; *P* = 0.934
Tyre tracks	4 (0.5)	2 (0.4)	χ^2^ = 0.005; *df* = 1; *P* = 0.945
Brick pits	89 (11.6)	40 (8.6)	χ^2^ = 1.023; *df* = 1; *P* = 0.312
Construction ditches	22 (2.9)	25 (5.4)	**χ^2^ = 7.862; *df* = 1; *P* = 0.005**
Hoof prints	2 (0.3)	16 (3.4)	**χ^2^ = 24.41; *df* = 1; *P* < 0.001**
Ponds	14 (1.8)	10 (2.1)	χ^2^ = 0.546; *df* = 1; *P* = 0.460
Rain pools	48 (6.3)	27 (5.8)	χ^2^ = 0.070; *df* = 1; *P* = 0.792
Run-off from natural source	9 (1.2)	2 (0.4)	χ^2^ = 1.349; *df* = 1; *P* = 0.246
Water storage containers	10 (1.3)	25 (5.4)	**χ^2^ = 22.457; *df* = 1; *P* < 0.001**
Tree holes	1 (0.1)	0 (0.0)	χ^2^ = 0.532; *df* = 1; *P* = 0.466
Bathroom run-offs	13 (1.7)	3 (0.6)	χ^2^ = 1.848; *df* = 1; *P* = 0.174
Any place with water	1 (0.1)	0 (0.0)	χ^2^ = 0.532; *df* = 1; *P* = 0.466
No idea	2 (0.3)	0 (0.0)	χ^2^ = 1.065; *df* = 1; *P* = 0.302

HA = health animator; LSM = larval source management.

The χ^2^ tests indicate comparisons between participants from the general community and HAs+LSM committee members. *P* values in bold are significant at alpha = 0.05.

### Knowledge of mosquito control methods.

Concerning knowledge or experience with mosquito control methods, participants’ responses from the general community differed from those of HAs+LSM committee members (χ^2^ = 41.043; *df* = 4; *P* < 0.001). For example, 4.9% of the participants from the general community listed incorrect methods such as cleaning the house and clearing bushes. No HA+LSM committee member listed an incorrect method.

### Vector control.

Ninety-six percent of all respondents felt that mosquitoes could be controlled. Of those participants who believed otherwise, 5% and 0.6% were participants from the general community and HAs+LSM committee members, respectively. These differences were significant (χ^2^ = 6.984; *df* = 1; *P* = 0.008). Among the reason why some participants did not believe that mosquitoes can be controlled were 1) it is difficult to locate all mosquito larval habitats, 2) there are insufficient preventive measures available, 3) preventive measures are inefficient, and 4) the level of community involvement in vector control initiatives is never adequate.

### Perception of habitat creation and importance.

When asked about the preferred environment for mosquito breeding, 88.7% of participants from the general community, and 79.9% of the HAs+LSM committee members considered standing dirty water to be the preferred environment for mosquito breeding. The responses invited further questions prompting whether the standing water served any purposes in the communities. Table 6 summarizes the responses on habitat creation and importance to human activities. Regarding importance, 37.5% and 43% of participants from the general community and HAs+LSM committee members, respectively, considered the habitats important. Of those participants in the former group who attached importance to the habitats, 67.1%, 28.9%, and 4.1% related the habitats to domestic, agricultural, and brick-making purposes, respectively. As for the HAs+LSM committee members, slightly more than half (51%) of those who felt that the habitats were important related the importance to domestic purposes. Further, 46.1% and 2.9% of the HAs+LSM committee members associated the habitats with agricultural purposes and brick making, respectively. Overall perception about habitat importance did not differ significantly between the two respondent groups (χ^2^ = 1.495; *df* = 1; *P* = 0.222).

**Table 6:** Perception on larval habitat importance and creation by study participants from LSM villages

Characteristic	Numbers (and percentage) of responses		Statistic
	Participants from the general community	HAs+LSM committee members	
Habitat importance			χ^2^ = 1.495; *df* = 1; *P* = 0.222
Total responses	146	104	
Domestic purposes	98 (67.1)	53 (51.0)	χ^2^ = 0.018; *df* = 1; *P* = 0.892
Agricultural purposes	42 (28.8)	48 (46.1)	**χ^2^ = 16.053; *df* = 1; *P* < 0.001**
Brick-making purposes	6 (4.1)	3 (2.9)	χ^2^ = 0.007; *df* = 1; *P* = 0.933
Habitat creation			**χ^2^ = 5.476; *df* = 1; *P* = 0.019**
Total responses	155	113	
Domestic purposes	73 (47.1)	28 (24.8)	χ^2^ = 2.688; *df* = 1; *P* = 0.101
Agricultural purposes	23 (14.8)	35 (31.0)	**χ^2^ = 19.100; *df* = 1; *P* < 0.001**
Brick-making purposes	59 (38.1)	50 (44.2)	**χ^2^ = 7.726; *df* = 1; *P* = 0.005**

HA = health animator; LSM = larval source management.

The χ^2^ tests indicate comparisons between participants from the general community and HAs+LSM committee members. *P* values in bold are significant at alpha = 0.05.

### Factors influencing motivation and participation in LSM.

Multivariate binary logistic regression analysis associated correct knowledge of *Bti* as a mosquito control tool in participants from the general community with reported motivation in LSM activities (Wald = 0.253; *df* = 1; *P <* 0.001). For HAs+LSM committee members, motivation was associated with the ability to recognize mosquito larvae (Wald = 9.841; *df* = 1; *P =* 0.002).

Concerning participation in the LSM activities, binary logistic regression using the same set of variables as in the above associated participation by participants from the general community with knowledge of mosquito aquatic habitats (Wald = 5.057; *df* = 1; *P =* 0.025) and knowledge of *Bti* as a mosquito control tool (Wald = 20.286; *df* = 1; *P <* 0.001). For the HAs+LSM committee members, participation in LSM activities was driven by their ability to recognize mosquito larvae (Wald = 11.55; *df* = 1; *P =* 0.001).

## DISCUSSION

Larviciding with *Bti* by trained community members (i.e., LSM committees) was effective in reducing larval densities within water bodies as evidenced by lower densities during post-spray surveys than pre-spray surveys. When compared with non-LSM villages, the community-led LSM did not, however, contribute significantly to reductions in anopheline larval densities in intervention villages, although larval densities in LSM villages decreased over time. The LSM committee members and HAs exhibited overall higher motivation and participation in the LSM activities. The ability to recognize mosquito larvae in water bodies, the knowledge about mosquito aquatic habitats and about *Bti* as a mosquito control tool positively influenced both motivation and participation in community-led LSM.

Contrary to our hypothesis, no differences in anopheline larval densities were observed between non-LSM villages and LSM villages during pre-spray surveys. The comparison of pre-spray survey densities in LSM villages with the densities in non-LSM villages was based on the assumption that consistent and repeated weekly applications of *Bti* would induce longitudinal reductions in the anopheline larval densities and that the effects would be reflected even during subsequent pre-spray surveys. Given that larval densities in both LSM and non-LSM villages were low during the study period, this absence of differences in larval densities is likely due to 1) the high coverage after mass distribution of long-lasting insecticidal nets in 2016 in the study villages, which may have suppressed vector populations overall,^46^^,^^47^ and 2) the unusually low precipitation experienced during the study period, which reduced the number of habitats containing water and also larval populations, also in the non-LSM villages. Indeed, low adult mosquito densities over the same period in the study area were reported.^48^ A third possibility may be the result of a “spillover effect” of the cluster randomized trial design: relatively short distances between LSM and non-LSM villages^42^ may have allowed mosquitoes to fly between the villages resulting in contamination of the LSM villages. However, although anopheline mosquitoes can fly further than the 800-m buffers established for this study with the aid of wind,^49^ most mosquitoes likely remain close to their larval habitats and houses, where suitable hosts reside.^50^

Our results from the KAP survey indicate that the majority of participants from the LSM villages had sufficient knowledge about people most at risk of malaria, its spread, vector larval habitats, and control efforts. It was also clear that, according to these results, the community realized that vector control is possible. Despite this knowledge, there was less participation by members of the community in the habitat draining and filling activities than by HAs+LSM committee members. One of the reasons for not removing water bodies may have been the important functions these habitats served, as also revealed by our KAP survey. In this study, the five most mentioned habitat types were human-made, and most of them served domestic and agricultural purposes. Similar conflicting interests were observed in Kenya,^51^^,^^52^ where communities were not willing to remove sites they deemed important for their livelihoods. Apart from the need for deliberate efforts by the government to make *Bti* more accessible by communities, other alternative larval control interventions that reduce larval densities without removing the water sources need to be explored as well.^53^

Motivation and participation in the community-led LSM were associated with ability to recognize mosquito larvae, which probably increased understanding about the risk of malaria and the need to manage the breeding sites. The LSM curriculum developed by the MMP for LSM committees emphasized understanding of the malaria topic and also gaining leadership skills for proper execution and management of the intervention. It was clear that the tailored trainings given to the LSM committee members instilled both knowledge of malaria risk and also a sense of ownership of the intervention. This, to a greater extent, set the committee members apart from the other members of the community. It could thus be suggested that for successful implementation of community-led disease control initiatives, investment should be directed toward training selected groups of people to become “local experts,” with greater understanding of their problems because this would promote local leadership and ownership of the initiative.

## CONCLUSIONS

Participation in LSM is dependent on community knowledge about vector biology and control and about roles assigned to individuals in the intervention. Although effective in increasing a community’s knowledge about malaria and its risk factors and control methods, community-led LSM did not significantly contribute to reductions in anopheline larval densities in the setting of our study where core malaria control interventions had been scaled up.

## Supplemental files


Supplemental materials

## References

[1] Fillinger U Lindsay SW, 2011. Larval source management for malaria control in Africa: myths and reality. Malar J 10: 353. 22166144 10.1186/1475-2875-10-353 PMC3273449

[2] Derua YA Kweka EJ Kisinza WN Githeko AK Mosha FW, 2019. Bacterial larvicides used for malaria vector control in sub-Saharan Africa: review of their effectiveness and operational feasibility. Parasit Vectors 12: 1–18. 31470885 10.1186/s13071-019-3683-5 PMC6716942

[3] Tusting LS Thwing J Sinclair D Fillinger U Gimnig J Bonner KE Bottomley C Lindsay SW, 2013. Mosquito larval source management for controlling malaria. Cochrane Database Syst Rev 8: CD008923. 10.1002/14651858.CD008923.pub2 PMC4669681 23986463

[4] Fillinger U Ndenga B Githeko A Lindsay SW, 2009. Integrated malaria vector control with microbial larvicides and insecticide-treated nets in western Kenya: a controlled trial. Bull World Health Organ 87: 655–665. 19784445 10.2471/BLT.08.055632 PMC2739910

[5] Worrall E Fillinger U, 2011. Large-scale use of mosquito larval source management for malaria control in Africa: a cost analysis. Malar J 10: 338. 22067606 10.1186/1475-2875-10-338 PMC3233614

[6] Mukabana WR , 2006. Ecologists can enable communities to implement malaria vector control in Africa. Malar J 5: 9. 16457724 10.1186/1475-2875-5-9 PMC1409792

[7] Majambere S , 2010. Is mosquito larval source management appropriate for reducing malaria in areas of extensive flooding in the Gambia? A cross-over intervention trial. Am J Trop Med Hyg 82: 176–184. 20133989 10.4269/ajtmh.2010.09-0373 PMC2813154

[8] Gu W Utzinger J Novak RJ, 2008. Habitat-based larval interventions: a new perspective for malaria control. Am J Trop Med Hyg 78: 2–6. 18187774

[9] Dambach P Schleicher M Stahl HC Traoré I Becker N Kaiser A Sié A Sauerborn R, 2016. Routine implementation costs of larviciding with *Bacillus thuringiensis* israelensis against malaria vectors in a district in rural Burkina Faso. Malar J 10: 380. 10.1186/s12936-016-1438-8 PMC4957841 27449023

[10] Maheu-Giroux M Castro MC, 2013. Impact of community-based larviciding on the prevalence of malaria infection in Dar es Salaam, Tanzania. PLoS One 8: e71638. 23977099 10.1371/journal.pone.0071638 PMC3743749

[11] Karunamoorthi K, 2011. Vector control: a cornerstone in the malaria elimination campaign. Clin Microbiol Infect 17: 1608–1616. 21996100 10.1111/j.1469-0691.2011.03664.x

[12] Zahiri NS Mulla MS, 2005. Non-larvicidal effects of *Bacillus thuringiensis* israelensis and *Bacillus sphaericus* on oviposition and adult mortality of *Culex* quinquefasciatus Say (Diptera: Culicidae). J Vector Ecol 30: 155–162. 16007971

[13] Walker K Lynch M, 2007. Contributions of Anopheles larval control to malaria suppression in tropical Africa: review of achievements and potential. Med Vet Entomol 21: 2–21. 17373942 10.1111/j.1365-2915.2007.00674.x

[14] Boyce R Lenhart A Kroeger A Velayudhan R Roberts B Horstick O. *Bacillus thuringiensis* israelensis (Bti) for the control of dengue vectors: systematic literature review. Trop Med Int Heal 18: 564–577. 10.1111/tmi.12087 23527785

[15] World Health Organization , 2013. Larval source management: a supplementary measure for malaria control: an operational manual. Outlooks Pest Manag 25: 41–43.

[16] Fillinger U Lindsay SW, 2006. Suppression of exposure to malaria vectors by an order of magnitude using microbial larvicides in rural Kenya. Trop Med Int Health 11: 1629–1642. 17054742 10.1111/j.1365-3156.2006.01733.x

[17] Fillinger U , 2008. A tool box for operational mosquito larval control: preliminary results and early lessons from the Urban Malaria Control Programme in Dar es Salaam, Tanzania. Malar J 7: 20. 18218148 10.1186/1475-2875-7-20 PMC2259364

[18] Gill SS Singh GJP Hornung JM, 1987. Cell membrane interaction of *Bacillus thuringiensis* subsp. israelensis cytolytic toxins. Infect Immun 55: 1300–1308. 3570465 10.1128/iai.55.5.1300-1308.1987 PMC260505

[19] Bravo A Gill SS Soberón M, 2007. Mode of action of *Bacillus thuringiensis* Cry and Cyt toxins and their potential for insect control. Toxicon 49: 423–435. 17198720 10.1016/j.toxicon.2006.11.022 PMC1857359

[20] Schnepf E Crickmore N Van Rie J Lereclus D Baum J Feitelson J Zeigler DR Dean DH, 1998. *Bacillus thuringiensis* and its pesticidal crystal proteins. Microbiol Mol Biol Rev 62: 775–806. 9729609 10.1128/mmbr.62.3.775-806.1998 PMC98934

[21] Poopathi S Abidha S, 2013. Mosquitocidal bacterial toxins (*Bacillus sphaericus* and *Bacillus thuringiensis* serovar israelensis): mode of action, cytopathological effects and mechanism of. J Physiol Pathophysiol 1: 22–38.

[22] Thomas WE Ellar DJ, 1983. Mechanism of action of *Bacillus thuringiensis* var israelensis insecticidal δ-endotoxin. FEBS Lett 154: 362–368. 6832375 10.1016/0014-5793(83)80183-5

[23] Wirth MC Park HW Walton WE Federici BA, 2005. Cyt1A of *Bacillus thuringiensis* delays evolution of resistance to Cry11A in the mosquito *Culex* quinquefasciatus. Appl Environ Microbiol 71: 185–189. 15640186 10.1128/AEM.71.1.185-189.2005 PMC544219

[24] Becker N Charles JF Delécluse A Roux CNL Entomopathogenic Bacteria: From Laboratory to Field Application. Dordrecht, The Netherlands: Springer, 383–398.

[25] Gould F, 1994. *Bacillus thuringiensis*, an environmental biopesticide: theory and practice. Philip F. Entwistle, Jenny S. Cory, Mark J. Bailey, Stephen Higgs [book review]. Q Rev Biol 69: 545–546.

[26] Olalubi OA, 2016. Promoting larval source management as a vital supplemental addendum and more likely cost-effective approach for malaria vector control in Nigeria. J Prev Infect Control 2: 1–6.

[27] Gowelo S McCann RS Koenraadt CJM Takken W Van Den Berg H Manda-Taylor L, 2020. Community factors affecting participation in larval source management for malaria control in Chikwawa District, Southern Malawi. Malar J 19: 1–11. 32487233 10.1186/s12936-020-03268-8 PMC7265157

[28] Kibe LW Mbogo CM Keating J Molyneux S Githure JI Beier JC, 2006. Community based vector control in Malindi, Kenya. Afr Health Sci 6: 240–246. 17604514 10.5555/afhs.2006.6.4.240 PMC1832065

[29] Chaki PP Dongus S Fillinger U Kelly A Killeen GF, 2011. Community-owned resource persons for malaria vector control: enabling factors and challenges in an operational programme in Dar es Salaam, United Republic of Tanzania. Hum Resour Health 9: 21. 21955856 10.1186/1478-4491-9-21 PMC3204271

[30] Atkinson JA Vallely A Fitzgerald L Whittaker M Tanner M, 2011. The architecture and effect of participation: a systematic review of community participation for communicable disease control and elimination. Implications for malaria elimination. Malar J 10: 225. 21816085 10.1186/1475-2875-10-225 PMC3171376

[31] Chilaka MA, 2005. Ascribing quantitative value to community participation: a case study of the Roll Back Malaria (RBM) initiative in five African countries. Public Health 119: 987–994. 16188287 10.1016/j.puhe.2005.08.010

[32] Chaki PP Kannady K Mtasiwa D Tanner M Mshinda H Kelly AH Killeen GF, 2014. Institutional evolution of a community-based programme for malaria control through larval source management in Dar es Salaam, United Republic of Tanzania. Malar J 13. 245. 24964790 10.1186/1475-2875-13-245 PMC4082415

[33] Van Den Berg H Von Hildebrand A Ragunathan V Das PK, 2007. Reducing vector-borne disease by empowering farmers in integrated vector management. Bull World Health Organ 85: 561–566. 17768506 10.2471/BLT.06.035600 PMC2636366

[34] Vanek MJ Shoo B Mtasiwa D Kiama M Lindsay SW Fillinger U Kannady K Tanner M Killeen GF, 2006. Community-based surveillance of malaria vector larval habitats: a baseline study in urban Dar es Salaam, Tanzania. BMC Public Health 6: 1–8. 16776829 10.1186/1471-2458-6-154 PMC1534019

[35] McCann RS , 2021. The effect of community-driven larval source management and house improvement on malaria transmission when added to the standard malaria control strategies in Malawi: a cluster-randomized controlled trial. Malar J 20: 1–24. 34022912 10.1186/s12936-021-03769-0 PMC8140568

[36] Ingabire CM , 2017. Community-based biological control of malaria mosquitoes using *Bacillus thuringiensis* var. israelensis (Bti) in Rwanda: community awareness, acceptance and participation. Malar J 16: 1–13. 28974204 10.1186/s12936-017-2046-y PMC5627396

[37] Van Den Berg H , 2018. Community-based malaria control in southern Malawi: a description of experimental interventions of community workshops, house improvement and larval source management. Malar J 17: 266. 30012147 10.1186/s12936-018-2415-1 PMC6048888

[38] McCann RS , 2017. Assessment of the effect of larval source management and house improvement on malaria transmission when added to standard malaria control strategies in southern Malawi: study protocol for a cluster-randomised controlled trial. BMC Infect Dis 17: 639. 28938876 10.1186/s12879-017-2749-2 PMC5610449

[39] Whittaker M Smith C, 2015. Reimagining malaria: five reasons to strengthen community engagement in the lead up to malaria elimination. Malar J 14: 1–6. 26474852 10.1186/s12936-015-0931-9 PMC4608300

[40] Heggenhougen HK Hackethal V Vivek P, UNDP/World Bank/WHO Special Programme for Research and Training in Tropical Diseases, 2003. The Behavioural and Social Aspects of Malaria and Its Control. Document No. TDR/STR/SE:214. Geneva, Switzerland: World Health Organization.

[41] Kabaghe AN Chipeta MG McCann RS Phiri KS Van Vugt M Takken W Diggle P Terlouw AD, 2017. Adaptive geostatistical sampling enables efficient identification of malaria hotspots in repeated cross-sectional surveys in rural Malawi. PLoS One 12: 1–14. 10.1371/journal.pone.0172266 PMC5308819 28196105

[42] McCann RS van den Berg H Takken W Chetwynd AG Giorgi E Terlouw DJ Diggle PJ, 2018. Reducing contamination risk in cluster-randomized infectious disease-intervention trials. Int J Epidemiol 47: 2015–2024. 30376050 10.1093/ije/dyy213

[43] Gowelo S Chirombo J Koenraadt CJM Mzilahowa T van den Berg H Takken W McCann RS, 2020. Characterisation of anopheline larval habitats in southern Malawi. Acta Trop 210: 105558. 32485166 10.1016/j.actatropica.2020.105558 PMC7673143

[44] Malenga T Kabaghe AN Manda-Taylor L Kadama A McCann RS Phiri KS van Vugt M van den Berg H, 2017. Malaria control in rural Malawi: implementing peer health education for behaviour change. Global Health 13: 84. 29157284 10.1186/s12992-017-0309-6 PMC5694909

[45] Service MW. Mosquito Ecology: Field Sampling Methods, 2nd edition. London, UK: Elsevier Applied Science.

[46] National Malaria Control Programme (NMCP) and ICF, 2018. Malawi Malaria Indicator Survey 2017. Lilongwe, Malawi, and Rockville, Maryland, USA: NMCP and ICF.

[47] National Statistical Office (NSO) [Malawi] and ICF, 2017. Malawi Demographic and Health Survey 2015-16. Zomba, Malawi, and Rockville, Maryland, USA: NSO and ICF.

[48] McCann RS , 2021. The effect of community-driven larval source management and house improvement on malaria transmission when added to the standard malaria control strategies in Malawi: a cluster-randomized controlled trial. Malar J 20: 1–160. 34022912 10.1186/s12936-021-03769-0 PMC8140568

[49] Manoukis NC Baber I Diallo M Sogoba N Ribeiro JMC, 2011. Seasonal climate effects anemotaxis in newly emerged adult Anopheles gambiae Giles in Mali, West Africa. PLoS One 6: e26910. 22114663 10.1371/journal.pone.0026910 PMC3217951

[50] Guerra CA , 2014. A global assembly of adult female mosquito mark-release-recapture data to inform the control of mosquito-borne pathogens. Parasit Vectors 7: 1–15. 24946878 10.1186/1756-3305-7-276 PMC4067626

[51] Imbahale SS Githeko A Mukabana WR Takken W, 2012. Integrated mosquito larval source management reduces larval numbers in two highland villages in western Kenya. BMC Public Health 12: 362. 22607227 10.1186/1471-2458-12-362 PMC3433356

[52] Imbahale SS Fillinger U Githeko A Mukabana WR Takken W, 2010. An exploratory survey of malaria prevalence and people’s knowledge, attitudes and practices of mosquito larval source management for malaria control in western Kenya. Acta Trop 115: 248–256. 20399739 10.1016/j.actatropica.2010.04.005

[53] Takken W Knols BGJ, 2009. Malaria vector control: current and future strategies. Trends Parasitol 25: 101–104. 19168392 10.1016/j.pt.2008.12.002

