# Supplementary material for: Community Participation in Habitat Management and Larviciding for the Control of Malaria Vectors in Southern Malawi
Source: Am J Trop Med Hyg. 2022 Nov 21;108(1):51–60. doi: 10.4269/ajtmh.21-1127 (PMC9833073; doi:10.4269/ajtmh.21-1127)
Supplement: Supplementary file 1 [file tpmd211127.SD1.pdf]

Supplementary material

Appendix A: Spin-the-bottle method for selecting larval habitats

Eligible habitats: any habitat the LSM committee is spraying with *Bti*, and which has a minimum depth along at least part of the edge that is less than 30cm.

Selection of Habitat 1

- Start at center of village.
- Spin a glass bottle on the ground.
- The first habitat (which the LSM committee is spraying with *Bti*) in the direction the bottle is pointing is Habitat 1 for larval density sampling.
- The habitat does not have to be a minimum distance from the center of the village.
- If there isn't a habitat (which the LSM committee is spraying with *Bti*) in that direction, spin the bottle again at the center of the village.

Selection of Habitat 2

- At location of Habitat 1, spin a glass bottle on the ground.
- The first habitat in the direction the bottle is pointing, *farther than 50m from Habitat 1*, is Habitat 2 for larval density sampling.
- If there isn't a habitat (which the LSM committee is spraying with *Bti*) in that direction farther than 50m from Habitat 1, then spin the bottle again at the location of Habitat 1.
- If there isn't a habitat (which the LSM committee is spraying with *Bti*) farther than 50m in any direction, then the first habitat in the direction the bottle is pointing is Habitat 2.

Selection of Habitat 3

- At location of Habitat 2, spin a glass bottle on the ground.
- The first habitat in the direction the bottle is pointing, *farther than 50m from the both Habitat 1 and Habitat 2*, is Habitat 3 for larval density sampling.

- If there isn’t a habitat (which the LSM committee is spraying with *Bti*) in that direction farther than 50m from Habitat 1 and Habitat 2, then spin the bottle again at the location of Habitat 2.
- If there isn’t a habitat (which the LSM committee is spraying with *Bti*) farther than 50m in any direction, then the first habitat in the direction the bottle is pointing is Habitat 3.

Note: If there are only 3 habitats, or fewer, sample at all of them. There would be no need for random selection in this case.

Appendix B: Semi-structured questionnaire for participants in the LSM arm of the study (English version)

**Village:**
**Demographic Features**
Age (years)
18-25
26 - 40
41 - 64
65+
Gender
Male
Female
Education
Illiterate
Primary
Secondary
Graduate & above
Main Source of Income
Employed
Agriculture
Business
Manual labour
None
**Knowledge of Malaria, Spread and Symptoms**

Do you have any knowledge of malaria?
Yes
No
Have you ever suffered from malaria? (diagnosed malaria)
Yes
No
Has anybody you know ever suffered from malaria? (diagnosed malaria)
Yes
No
What are the common symptoms of malaria? (multiple choice)
Fever
Nausea
Headache
Body aches
Vomiting
Shivering
Diarrhea
What are the common symptoms of malaria? (multiple choice)
Convulsion
No idea
Who do you perceive to be the most at risk for malaria? (open question)
In your opinion, how do people get malaria?
Mosquito bite
Fly bite
Witchcraft

Soaking in rain
Other (specify)
No idea
**Knowledge about malaria control**
Do you think it is possible to control mosquitoes?
Yes
No
If yes, how do you think mosquitoes can be controlled? (multiple choice)
Smoke
Mosquito coils
Mosquito spray
Fan
Covering of body with clothes
Mosquito net
Skin repellents
Cleaning house
Indoor Residual spraying
Removing standing water
Clearing of bushes
Other (specify)
No idea
If no, why do you think it is not possible to control mosquitoes? (open question)
What is the name of the malaria vector?
Female Anopheles
Male Anopheles

Culex
Other (specify)
No idea
**Knowledge about mosquito larvae**
Are you able to recognise mosquito larvae?
Yes
No
If yes, are you able to distinguish anopheline larvae from culicine?
Yes
No
**Knowledge about mosquito breeding**
In what type of environment do anopheline mosquitoes like to breed? (multiple choice)
Running dirty water
Garbage/Trash
Standing clean water
Standing dirty water
Running Clean water
Plants/ vegetation
Other (specify)
No idea
What are common aquatic habitats for anopheline mosquitoes? (multiple choice)
Pit-latrine
Rice paddies
Wells

Drainage Channels
Bore hole run-offs
Dams
Stream Beds
Freshwater marshes
Tire tracks
Brick pits
Hoof print aggregations
Ponds
Rain Pools
Run-offs from natural sources
Other (specify)
[Explanation of the correct breeding habitats of anophelines by the research assistant: E.g. clean standing water in sites like borehole run-offs, wells, etc]
**Creation and importance of mosquito breeding sites**
Do you think that these aquatic habitats are important for your livelihood?
Yes
No
Why do you think that these aquatic habitats are important for your livelihood? (open question)
Do you create these sites yourself?
Yes
No
For what purposes do you create these sites? (open question)

**Knowledge about mosquito larval control methods**
Eradication of breeding site of mosquito (multiple choice)
Draining
Filling up
Changing water in storage tanks
Others (specify)
No idea
Are you aware of *Bti* larviciding?
Yes
No
Negative effects of *Bti* on spray operators
Many
Few
None
No idea
Negative effects of *Bti* on livestock
Many
Few
None
No idea
Negative effects of *Bti* on crops
Many
Few
None

No idea
Negative effects of *Bti* on crop consumers and water users
Many
Few
None
No idea
Participation in LSM activities (Draining & filling)
Active
Less active
Not at All
Motivation in LSM activities (Draining & filling)
A lot
Little
None
**for LSM Committee members only**
Participation in *Bti* spraying
Active
Less Active
Not at All
Motivation in *Bti* spraying
A lot
Little
None
